# Assessing the Impact of Drought Stress on Hemp (*Cannabis sativa* L.) Fibers

**DOI:** 10.3390/ma17174198

**Published:** 2024-08-24

**Authors:** Edyta Kwiatkowska, Małgorzata Zimniewska, Wanda Różańska, Michał Puchalski, Patrycja Przybylska

**Affiliations:** 1 Department of Innovative Textile Technologies, Institute of Natural Fibers and Medicinal Plants—National Research Institute, 60-630 Poznan, Poland; malgorzata.zimniewska@iwnirz.pl (M.Z.); wanda.rozanska@iwnirz.pl (W.R.); patrycja.przybylska@iwnirz.pl (P.P.); 2 Division of Materials Science, Commodity Science and Textile Metrology, Textile Institute, Faculty of Material Technologies and Textile Design, Lodz University of Technology, 90-543 Lodz, Poland; michal.puchalski@p.lodz.pl

**Keywords:** hemp, fibers, drought stress, cellulose, hemicellulose, pectin, lignin, FTIR, WAXD, TGA

## Abstract

Drought can significantly impact fiber crop cultivation due to the plants’ specific water requirements and their extended vegetative period. The purpose of the research was to examine how drought stress affects the quality and chemical composition of hemp (*Cannabis sativa* L.) fibers. A three-year pot experiment was conducted in a plant growth facility, using controlled drought stress for hemp plants. Soil moisture levels were maintained at three levels, where 45% field water capacity was the control and 35% and 25% FWC were drought. A comprehensive suite of fiber quality characterization techniques, including linear density measurement, tenacity assessment, Fourier Transform Infrared Spectroscopy (FTIR), and Wide-Angle X-ray Diffraction (WAXD), was employed to evaluate the impact of drought stress on fiber properties. The chemical composition of hemp fibers was thoroughly analyzed, quantifying the content of cellulose, hemicellulose, pectin, and lignin. The findings indicate that drought conditions significantly influence linear density, wax and fat content, as well as the crystallinity of the fibers.

## 1. Introduction

Hemp (*Cannabis sativa* L.) belongs to the plant species in the family Cannabaceae. The Cannabis genus contains a single species—*Cannabis sativa* L. (hemp)—which encompasses both fiber hemp (*Cannabis sativa* L. var. *sativa*) and Indian hemp (*Cannabis sativa* L. var. *indica*) [1]. Hemp is among the most renowned and debated plant species globally due to its wide-ranging applications in both industry and medicine. The legality of cultivating, possessing, and using hemp varies greatly worldwide due to its potential psychoactive effects, especially in varieties containing high levels of THC. In some places, hemp cultivation is completely legal, while in others, it is only available for medicinal purposes, and in certain countries, it remains prohibited.

In the Common Catalogue of Varieties of Agricultural Plant Species, compiled by the European Commission, there are varieties of plants approved for circulation within the European Union. It comprised 102 hemp varieties and 4 conservation hemp varieties in 2023. These conservation varieties are essential plants naturally suited to local and regional conditions that are at risk of genetic erosion [2].

Interest in hemp-derived raw materials and hemp-based bioproducts is growing worldwide. The considerable ecological advantages of plants, coupled with their diverse range of raw material offerings, render industrial hemp attractive to sectors such as agriculture, medicine, food production, textiles, construction, and numerous other industries [3].

**Figure 1:**
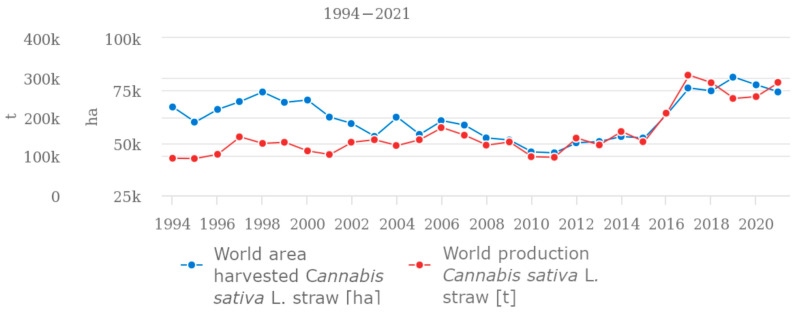
The global area and production of hemp straw [5].

In recent years, there has been an increase in the world production and world area harvested of *Cannabis sativa* L. straw (Figure 1), which is associated with a greater quantity of hemp fibers obtained. Increasingly, in clothing stores, in addition to linen products, one can find clothing made from hemp fibers. The positive antioxidant and antibacterial effects, especially of the Bialobrzeskie hemp fiber variety, provide the opportunity to create functional clothing that positively affects human skin [4].

Contemporary drought is becoming an increasingly serious global issue, particularly in the context of climate change. Many areas worldwide, especially in dry and semi-arid regions, are experiencing more pronounced effects of drought, including reduced agricultural yields, problems with access to water, and degradation of the natural environment. Hemp plants, especially those cultivated for fiber, can be significantly affected by drought. An adequate amount of water is essential for plants during growth and flowering stages. Drought induces plant stress, which can affect their resistance to pests and increase disease occurrence. As a result, hemp plant yields may be greatly reduced, leading to lower fiber quantities, which can negatively impact cultivation profitability. Drought can also affect the chemical composition of hemp plants, including the content of active compounds such as cannabinoids. Some studies suggest that stressful conditions like drought can influence cannabinoid production in hemp plants [6]. In combating drought, farmers often have to resort to additional measures such as artificial irrigation, which disturbs sustainability performance and increases costs. Therefore, drought poses a significant challenge for producers of hemp plants for fiber and for the entire agricultural sector. Solutions such as developing drought-resistant varieties, investing in water-efficient irrigation systems, and implementing sustainable agricultural practices can help manage the effects of drought on hemp plant cultivation and other agricultural crops.

The aim of current research was to investigate whether drought during the cultivation of hemp plant can affect the quality of the fibers and their chemical composition, potentially impacting subsequent processing of hemp fibers not only in the textile sector but also in composite development. The research is innovative because it explores a relatively uncharted area of agricultural science, particularly in the context of climate change. This research is significant as it provides crucial insights into how environmental stressors like drought can impact the performance and usability of hemp fibers in various industries, including textiles and construction. Understanding these effects can lead to the development of more resilient hemp varieties and sustainable cultivation practices, which are essential for maintaining fiber quality and ensuring the long-term viability of hemp as a versatile, eco-friendly resource.

## 2. Materials and Methods

### 2.1. Experimental Schedule

The study focused on Bialobrzeskie (*Cannabis sativa* L.) hemp variety, cultivated under controlled conditions with soil moisture levels at 25%, 35%, and 45% of the field water capacity (FWC). The experiment spanned three consecutive years: 2019, 2020, and 2021. The climate and soil composition remained consistent for all plants each year.

**Figure 2:**
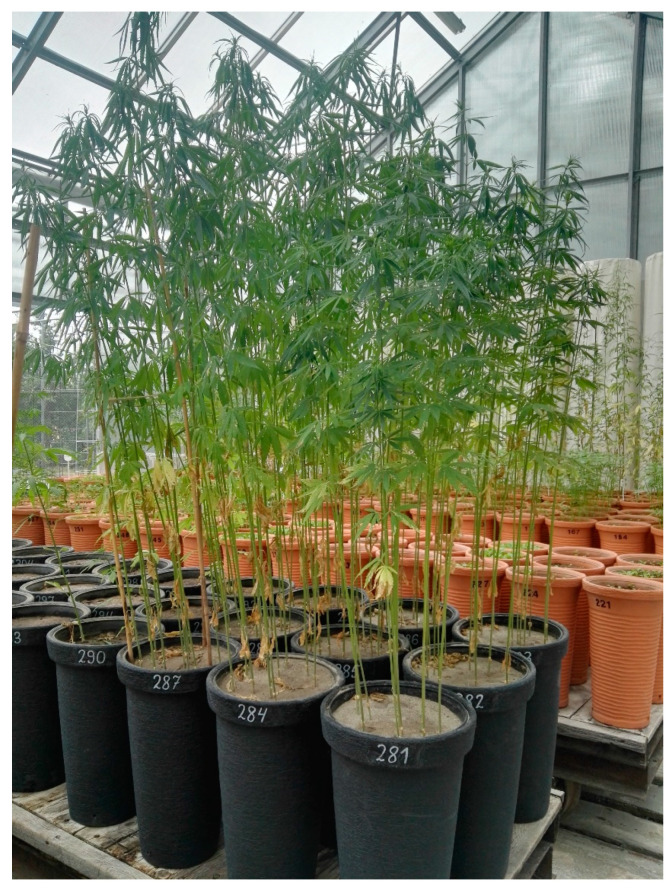
The 73rd day of hemp growth out of 153 days in 2019. Source: the authors’ materials, Edyta Kwiatkowska; Institute of Natural Fibres and Medicinal Plants—National Research Institute, Poland, 5 July 2019.

The experiment was conducted in a plant growth facility of the Institute of Natural Fibers and Medicinal Plants National Research Institute in Petkowo (52°12′40″ N, 17°15′31″ E). The setup included pots with a diameter of 40 cm and height of 60 cm, each replicated nine times (Figure 2). These pots were weighed and filled with field-sourced soil suitable for hemp crop rotation. Each pot was sown with 20 hemp seeds at a depth of 1 cm. Post-sowing, a 0.5 cm thick layer of quartz sand (400 g) was applied to the soil surface in each pot to prevent excessive drying and crusting. From sowing until the rapid growth phase (when plants reached 18–20 cm in height), soil moisture was maintained at an optimal 45% FWC. “Field water capacity” refers to the maximum water content the soil can hold after saturation and draining excess water.

Once the hemp plants achieved balanced growth, particularly during the early stages when they reached 5–6 cm in height, a thinning process began. Each pot was left with 10–12 healthy and evenly grown plants. Upon reaching 18–20 cm, indicating the start of rapid growth, controlled soil water deficits were introduced: 25% FWC in three pots, 35% FWC in another three pots, and the remaining three pots at the control level of 45% FWC. Symbols representing the hemp varieties under specific soil moisture conditions are shown in Table 1. Water supplementation was conducted using a weighing method based on soil moisture determination and FWC, measured using the Wanschaty method. The experiment was pesticide-free.

Hemp plants were harvested manually, pulling the plants from pots. After harvesting, the roots were trimmed, and excess leaves and seeds from the panicle were removed. After harvesting, hemp straw was spread evenly in the field and turned over periodically to ensure even straw retting. The natural retting process of the hemp straw in the field lasted 60 days. The degree of retting was assessed organoleptically by breaking the stalks and manually removing the woody parts (shives). If the straw broke easily and the fibers separated from the shives, the retting process was deemed complete. The straw was then harvested, dried, and mechanically processed to produce fibers.

The mechanical processing of the degummed straw was carried out on the following:

- A laboratory breaking machine (Czech Flax Machinery), suitable for hemp straw due to its stalk thickness;
- Laboratory turbine (Czech Flax Machinery).

### 2.2. Fiber Evaluation—Metrological Analysis

#### 2.2.1. Linear Density

The linear density of hemp fibers was analyzed using a gravimetric method based on the Polish Standard PN-EN ISO 1973:2011 [7]. Ten bundles of fibers, each 10 mm long and consisting of 100 pieces of hemp fibers, were separated. The bundles were weighed with an accuracy of 0.1 mg. The results were reported in tex.

#### 2.2.2. Tenacity

The tenacity of hemp fibers was tested according to the Polish Standard PN-P-04676:1986 [8]. The research was conducted using the STATIMAT ME tensile testing machine (Textechno, Mönchengladbach, Germany). The tenacity of 5 fiber bundles was determined. The distance between the clamps of the tensile tester was 3 mm, and the speed test was set to 10 mm/min.

### 2.3. Fiber Evaluation—Chemical Analysis

#### 2.3.1. Wax and Fat Content

The wax and fat content in hemp fibers was analyzed according to the Industry Standard BN-86/7501-10 [9]. The percentage content was determined by extracting wax and fat from two samples using petroleum ether (Sigma-Aldrich, St. Louis, MO, USA) in a Soxhlet apparatus for approximately 12 h, followed by weighing the dry residue.

#### 2.3.2. Cellulose Content

The analysis of cellulose content in hemp fibers was conducted according to the Polish Standard PN-92/50092 [10]. The analysis involved dissolving lignins and other substances in the fibers using a mixture of acetylacetone and dioxane, acidified with hydrochloric acid (Sigma-Aldrich).

#### 2.3.3. Hemicellulose Content

The hemicellulose content was determined following the Industry Standard BN-77/7529-02 [11]. The process involved dissolving hemicellulose in a 1% sodium hydroxide solution (Sigma-Aldrich), filtering, drying, and weighing the residue. The hemicellulose content was calculated from the mass loss of the sample.

#### 2.3.4. Pectin Content

The pectin content was determined using a gravimetric method developed at the Institute of Natural Fibres and Medicinal Plants National Research Institute. Pectins were dissolved in ammonium citrate (Sigma-Aldrich), precipitated with calcium chloride (Sigma-Aldrich), and the weight of the calcium pectinate precipitate was measured.

#### 2.3.5. Lignin Content

Lignin content in hemp fibers was measured according to the Industry Standard BN-86/7501-11 [12]. Hemp fibers were ground (using a RETSCH SM 100 Fritsch (Idar-Oberstein, Germany) cutting mill with a 1 mm sieve) with a mixture of concentrated sulfuric acid and orthophosphoric acid to dissolve cellulose, hemicellulose, and pectin. The insoluble lignin was filtered using a G4 filter crucible and dried to a constant weight.

### 2.4. FTIR-ATR Analysis

The Fourier Transform Infrared Attenuated Total Reflectance (FTIR-ATR) method was employed to identify and characterize organic and inorganic substances in the fibers. This technique measures the intensity of infrared radiation transmitted through the sample to determine its chemical composition. The analysis was conducted using the iS10 model from WatersTM | TA Instruments (New Castle, DE 19720, USA), equipped with the Smart iTX_ZnSe accessory. Each sample was subjected to 32 scans, with a resolution of 4 cm^−1^, covering the range of 600 to 4000 cm^−1^.

### 2.5. TGA-FTIR Analysis

Thermogravimetric analysis (TGA) combined with Fourier Transform Infrared Spectroscopy (FTIR) was performed to study the thermal stability and composition of the fibers. The TGA was conducted using a TA Instruments Analyzer Q50 (New Castle, DE, USA), heating approximately 15 mg of the sample from 30 to 700 °C at a rate of 10 °C/min in a nitrogen atmosphere at a constant gas flow rate of 90 mL/min.

During the TGA, the gases released were identified using FTIR, performed on a TA Instrument iZ10 model. The FTIR spectrum of the released gases was captured with 8 scans per second at a resolution of 4 cm^−1^ in the range of 600 to 4000 cm^−1^.

### 2.6. WAXD for Evaluation of Crystalline Structure

Wide-angle X-ray diffraction analysis of hemp fibers was conducted to analyze the supramolecular structure of the fibers in terms of crystalline phase using an X-ray diffractometer with the X’Pert Pro System (PAN Analytical). The radiation source was a copper anode X-ray tube with CuKα radiation wavelength (λ = 0.154 nm). The accelerating voltage was set to 40 kV, with an anode current of 30 mA. The X’Celector semiconductor detector was used for detection. Prior to measurements, hemp fibers were ground using a cutting mill SM 100 by RETSCH with a 1 mm sieve. Diffraction patterns were obtained in the 2θ range of 0–60° with a step size of 2° under continuous operation in a single repetition. The degree of crystallinity was estimated using WAXSFIT 1.0 software based on the Hindeleh–Johnson method [13].

### 2.7. The Statistical Analysis

The statistical analysis of the results was conducted using TIBCO Statistica^®^ 13.3 software (Poznań, Poland).

## 3. Results and Discussion

The analysis of the impact of drought on the quality of hemp fibers and their chemical composition has brought a series of significant observations crucial for understanding hemp plant reactions to drought stress. In this study, it was observed that extreme drought conditions exert a significant influence on various qualitative and chemical aspects of hemp fibers. Plants were intentionally subjected to drought stress by controlling their access to water. Soil moisture levels were maintained at 25%, 35%, and 45% FWC. Similar studies were conducted regarding the impact of drought on the quality of flax fibers at the same FWC level [14]; however, data concerning hemp fibers are lacking. The following research will contribute to expanding the knowledge of the effects of drought on the bast fiber group.

### 3.1. Mechanical Properties

**Figure 3:**
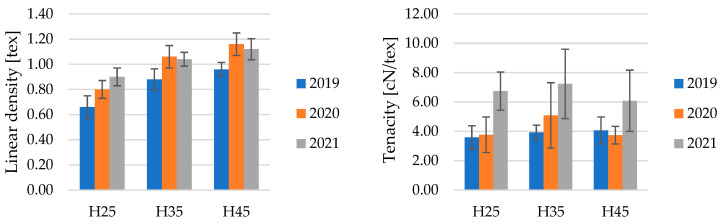
The linear density and tenacity of hemp fibers.

Linear density is one of the key parameters that influence the quality of fibers and significantly impact their application. Proper selection of linear density can be crucial for various industries using these fibers. The linear density of fibers greatly affects the linear density of yarn because an appropriate quantity of fibers should be present in the cross-section of the yarn. Thinner fibers used will result in yarn characterized by lower linear density, consequently leading to flat textile products made from these materials, achieving parameters suitable for the production of high-quality lightweight clothing items. Lower linear density allows for an increase in the number of fibers in the cross-section of the yarn, enabling the production of yarn with lower mass unevenness [15]. The results of the linear density (Figure 3) from three years of experimentation indicated that with the increase in soil moisture up to 45% FWC, the linear density also increased. This was further confirmed by the Kruskal–Wallis test (*p* = 0.0001), where, at the adopted level of significance (α = 0.05), it was indicated that the differences in the linear density of tested fibers were statistically significant.

The tenacity of fibers significantly influences yarn breaking strength, determining the mechanical properties of the yarn and the flat textile product it will be manufactured into. Therefore, the best possible values of this parameter are expected. The specific strength of fibers varied between individual years (Figure 3). Statistical analysis using the Kruskal–Wallis test (*p* = 0.5267) at the accepted significance level (α = 0.05) revealed no significant differences in the results, which is likely due to a large standard deviation of the results. Statistical tests were also conducted to check whether soil moisture significantly affects the tenacity by grouping the results from each year of the experiment. In each year, the results also turned out to be statistically insignificant.

Linear density is a crucial factor influencing the quality and application of fibers, particularly in textile production. It significantly affects the linear density of yarn, with thinner fibers leading to lighter, high-quality fabrics ideal for lightweight clothing. The study found that increasing soil moisture up to 45% FWC increased fiber linear density, with statistically significant results, which does not have a positive effect on the quality of fibers intended for the production of thin, high-quality fabrics. However, fiber tenacity, which affects yarn strength, showed no significant variation across different years, likely due to large standard deviations.

### 3.2. Chemical Analysis

Chemical analyses of natural fibers, including hemp fibers, aim to provide detailed information about their chemical composition. This information is crucial for various fields, including textile, pharmaceutical, construction, and others. Chemical analyses of hemp fibers have allowed for the determination of various components such as cellulose, hemicellulose, lignin, pectin, wax, and fat. Understanding the chemical composition of hemp fibers is fundamental for assessing their quality and suitability for various applications. Chemical analyses of hemp fibers enable a better understanding of their physical, mechanical, and biological properties, which is essential for further development and applications of this plant in various industrial and scientific sectors. The chemical composition of bast fibers varies depending on the plant and within the plant. It also varies depending on plant maturity, determined by harvesting time, fiber source, and applied method of fiber extraction [16].

#### 3.2.1. Wax and Fat Content

Wax and fat substances in hemp fiber serve several important functions, both in the context of the hemp plant itself and in industrial applications. Wax and fat substances play a role in creating a hydrophobic layer on the surface of the hemp plant, helping to minimize water loss through transpiration. This is particularly significant in dry conditions when plants need to effectively manage water availability. Additionally, wax serves a protective function, hindering pests’ access to the plant [17]. The significance of these substances in the textile industry is noteworthy because wax can act as a natural lubricant, facilitating the mechanical processing of hemp fibers during the production of various products.

**Figure 4:**
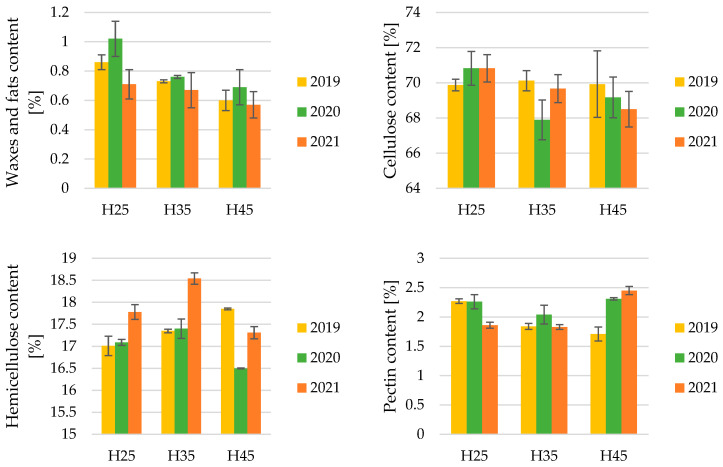

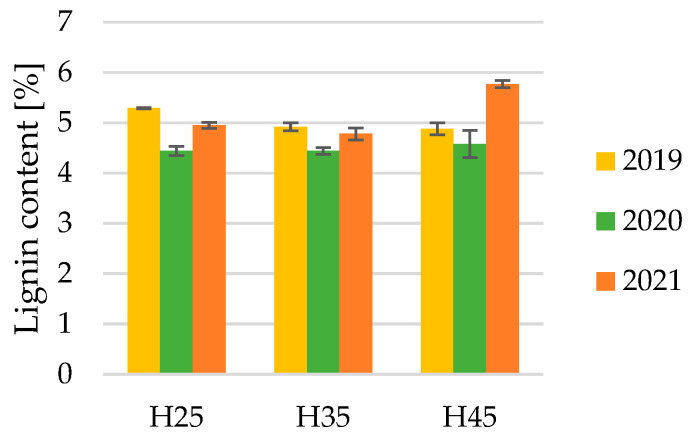
Chemical analyses of hemp fibers.

Research results have shown that as soil moisture increases, the content of wax and fat substances in the fiber decreases (Figure 4). The results of the Kruskal–Wallis test (*p* = 0.0177), with a significance level set at α = 0.05, indicated the statistical significance of the research results. With increasing drought, the amount of wax and fat substances in the fiber increases.

#### 3.2.2. Cellulose Content

Cellulose is a key component of hemp fibers, imparting them with strength, elasticity, and various physical properties. Cellulose is the main constituent of the plant’s cell wall, providing structural strength to the hemp fiber. In the cell wall, cellulose exists in two conformations: amorphous and crystalline [18]. The high degree of crystallinity of fibers influences their specific strength. A higher concentration of cellulose fibers enhances the economic advantage of extracting fibers. Additionally, fibers with elevated cellulose levels find wide-ranging uses across various applications [19].

The impact of drought on the cellulose content in fibers was examined (Figure 4). The results of the one-way analysis of variance (ANOVA) F-test (*p* = 0.1002), with a significance level set at α = 0.05, indicate no significant differences in the cellulose content in hemp fibers across varying soil moisture levels during plant growth. This suggests that drought does not significantly affect the cellulose content in hemp fibers. Statistical tests were also conducted to check whether soil moisture significantly affects the cellulose content by grouping the results from each year of the experiment. In each year, the results also turned out to be statistically insignificant.

#### 3.2.3. Hemicellulose Content

Hemicellulose consists of polysaccharides composed of a combination of furanose and pyranose sugars, such as glucose, mannose, xylose, galactose, rhamnose, and arabinose. Compared to crystalline cellulose, hemicellulose is a branched polysaccharide exhibiting various side chains, resulting in a non-crystalline structure. Hemicellulose is characterized by high hydrophilicity [20]. In terms of mechanical properties, hemicellulose does not contribute to the stiffness or strength of fibers [21].

The results of the study on the hemicellulose content in fibers across varying soil moisture levels (Figure 4), along with the statistical analysis using the one-way analysis of variance (ANOVA) F-test (*p* = 0.2095), with a significance level set at α = 0.05, indicate no significant differences. This means that drought does not significantly affect the hemicellulose content in hemp fibers. Statistical tests were also conducted to check whether soil moisture significantly affects the hemicellulose content by grouping the results from each year of the experiment. In each year, the results also turned out to be statistically insignificant.

#### 3.2.4. Pectin Content

The flexibility of fibrous plants is conferred by pectin. Additionally, pectin binds fibers into bundles. Because pectin undergoes degradation, it reduces the overall strength of the fiber, which is undesirable. Therefore, fibers with lower pectin content are characterized by better quality [17].

The results of the study on the pectin content in fibers across varying soil moisture levels (Figure 4), along with the statistical analysis using the Kruskal–Wallis test (*p* = 0.2024), with a significance level set at α = 0.05, indicate no significant differences in the research results. This means that drought does not significantly affect the pectin content in hemp fibers. Statistical tests were also conducted to check whether soil moisture significantly affects the pectin content by grouping the results from each year of the experiment. In each year, the results also turned out to be statistically insignificant.

#### 3.2.5. Lignin Content

Lignin provides increased stiffness to plants as they age due to its properties: high carbon content and moderate amounts of hydrogen [17]. An increase in lignin content in fibers negatively affects their quality, leading to a decrease in specific strength, elasticity, and fiber divisibility [22].

The results of the study on the lignin content in fibers across varying soil moisture levels (Figure 4), along with the statistical analysis using the one-way analysis of variance (ANOVA) F-test (*p* = 0.3429), with a significance level set at α = 0.05, indicate no statistical significance in the results. This means that drought does not significantly affect the lignin content in hemp fibers. Statistical tests were also conducted to check whether soil moisture significantly affects the lignin content by grouping the results from each year of the experiment. In each year, the results also turned out to be statistically insignificant.

The chemical composition of hemp fibers is crucial for their application in various industries. The study examined the impact of soil moisture on the content of wax, fat, cellulose, hemicellulose, pectin, and lignin in hemp fibers. The results showed that increased soil moisture leads to a decrease in wax and fat content, which is statistically significant. However, soil moisture does not significantly affect the content of cellulose, hemicellulose, pectin, or lignin in the fibers.

### 3.3. FTIR-ATR Analysis

The FTIR-ATR spectrophotometric analysis was conducted to elucidate the molecular structure of the surface layer of hemp fibers. The infrared spectra of the examined fibers were utilized to detect absorbance bands corresponding to the vibrations in specific functional groups, including O-H, C=O, C=C, COO, C-H, CH2, CH3, and COC originating from chemical constituents within the fiber [23]. The analysis of drought intensity in a specific year did not yield significant differences in the occurrence of peaks within a particular wave number range (Table 2). A detailed figure of FTIR-ATR spectra of hemp fibers can be found in Supplementary Figure S1. Only minor shifts in these peaks were observed, potentially attributable to vibrations of similar functional groups. Kwiatkowska et al. [24] conducted a similar FTIR-ATR study on flax fibers that were subjected to drought stress. The IR spectra of flax fibers and hemp fibers are very similar, with slight shifts. The drought did not significantly affect the results.

Spectrophotometric separation of individual chemical compounds within the fiber is unattainable due to the existence of identical functional groups across various components of the fiber. The analysis of fiber spectra spanning all experimental years and levels of FWC enabled the identification of shared bands within the wavelength range (Table 2).

The FTIR-ATR spectrophotometric analysis of hemp fibers was conducted to investigate their molecular structure by identifying specific functional groups through infrared spectra. The study found no significant differences in the absorbance peaks of these functional groups due to drought intensity, with only minor shifts observed, indicating resilience in the fiber’s chemical composition. Minor peak shifts in the infrared spectra were observed; these are likely due to similar functional group vibrations rather than drought effects. This suggests that drought conditions do not substantially alter the molecular structure of hemp fibers.

### 3.4. TGA-FTIR Analysis

Thermal decomposition tests revealed that decomposition for all tested fiber samples occurred within the temperature range of 220 to 540 °C (Table 3). The TGA curves indicated a single-step decomposition process for all fibers, while the DTG derivative analysis identified three distinct sub-stages of thermal degradation. The first sub-stage is visible in the range of 50–120 °C, the second in the range of 220–300 °C, and the third in the range of 300–420 °C. The results mentioned above are in agreement with the literature where, as reported by many authors [25,26,27,28,29], four sub-stages are distinguished during the thermal decomposition of flax or hemp fiber under pyrolysis conditions, which is related to the chemical composition of the fiber. According to the authors [25,26,27,28,29], the first stage is attributed to the evaporation of adsorbed water, the second to the degradation of hemicellulose, the third to the degradation of cellulose, and the fourth to the degradation of lignin. The last sub-stage is not clearly visible on the basis of the DTG curve, but according to Benıtez-Guerrero et al. [26] and Poletto et al. [28], it occurs in the temperature range of 410 °C to 600 °C, and according to Kim et al. [29], this process is slow and starts at a temperature of 250 °C.

Pyrolysis tests indicated that the thermal decomposition characteristics of hemp samples grown under different soil moisture levels were very similar, with negligible differences in thermograms and solid residue amounts after pyrolysis. The maxima of the DTG curves were also in similar temperature ranges. Statistical analysis using TIBCO Statistica^®^ 13.3 confirmed these findings. Statistical tests were carried out by checking whether soil moisture had a statistically significant effect on the parameter in question, with no breakdown by year of experiment and with a breakdown by year grouping the results of a given year. Statistical analysis of the thermal decomposition results showed an effect of soil moisture on the decomposition temperature of the main fiber component (DTG peak), which was carried out using a one-factor analysis of variance (*p* = 0.0083) at the accepted level of significance (α = 0.05). The study confirmed a significant relationship only for the year 2019, where the decay temperature was shown to decrease with increasing soil moisture (Table 3). For the other years, the results obtained were not statistically significant. A significant relationship was also found for the effect of soil moisture on the value of the temperature at 60% loss of fiber mass in pyrolysis (*p* = 0.0345), for which this temperature (T_60%_) was shown to decrease with increasing soil moisture. The above relationship was shown for tests in 2019 and 2021.

The statistical analysis of the results of the thermal decomposition of the fibers, using the Kruskal–Wallis test, showed no significant differences.

At the same time, Fourier Transform Infrared (FTIR) identification of the gases released was carried out to study the thermal decomposition of the fibers. The spectra were analyzed based on the four sub-stages of fiber degradation, i.e., water evaporation, hemicellulose, cellulose, and lignin degradation [25,26,27,28,29]. The spectra obtained for the decomposition temperatures of 100, 300, 365, and 450 °C corresponding to the above sub-stages were analyzed.

Detailed analysis of the spectra in selected temperature ranges allowed the identification of the gases released during fiber pyrolysis (Table 4). Detailed figures can be found in the Supplementary Figures S2–S4. Investigations have shown that in the first sub-stage discussed, water is evaporated. The spectra at a temperature of 100 °C show characteristic bands stretching the OH bonds at wavelengths of 3737 cm^−1^ and 1507 cm^−1^. However, the visible bands at 2362 cm^−1^ and 670 cm^−1^ are due to residual carbon dioxide present in the sample chamber and in the air. This can be explained by the fact that, despite the nitrogen purging, there are traces of carbon dioxide in the chamber, as shown in the spectrum for all samples.

The analysis of the spectra for the second and third substages, i.e., for temperatures of 300 °C and 365 °C, allowed the identification of the evolution of the same gases for both substages as:

- Water vapor:
  ○ OH bond stretching bands: 3740–3733 cm^−1^ and 1507 cm^−1^.
- Carbon dioxide:
  ○ CO bond stretching bands: 2362–2361 cm^−1^ and 673–670 cm^−1^.
- Carbon monoxide:
  ○ CO bond band: 2195–2183 cm^−1^.
- Acetic acid and formic acid:
  ○ OH bond stretching vibrations: 3571–3567 cm^−1^;
  ○ C=O bond stretching vibrations (carbonyl group): 1773–1745 cm^−1^;
  ○ C-O bond stretching vibrations (acids): 1180–1077 cm^−1^;
  ○ -CH_3_ bond stretching vibrations:2983–2976 cm^−1^.
- Formaldehyde:
  ○ CH bond vibration bands (aldehyde group): 2834–2724 cm^−1^;
  ○ CH_2_ vibration bands: 2934–2904 cm^−1^;
  ○ C=O bond vibration (carbonyl group): 1773–1745 cm^−1^.

However, analysis of the spectra for the decomposition temperature of 450 °C allowed the identification of gases such as:

- Water vapor, stretching OH bond visible at 3737–3736 cm^−1^;
- Carbon dioxide, stretching CO bond visible at 2362–2361 cm^−1^ and at 673–670 cm^−1^;
- Carbon monoxide, stretching CO bond visible at 2188–2182 cm^−1^;
- Formaldehyde, stretching CH bond at 2931–2928 cm^−1^ and C=O bond at 1755–1752 cm^−1^;
- Methane, for which CH_4_ bond stretching vibration bands: 3022–3020 cm^−1^.

Infrared studies indicated that the thermal decomposition of hemp fiber material under pyrolysis conditions mainly involves the release of carbon dioxide, organic compounds, water vapor, and, to a lesser extent, carbon monoxide. However, the release of organic compounds depends on the decomposition temperature, with the initial release of acetic acid, formic acid, and formaldehyde observed above 200 °C and the release of methane observed above 380 °C.

According to Urbańczyk [30], the thermal decomposition of lignocellulosic components produces water vapor, carbon dioxide, carbon monoxide, formic and acetic acids, methyl alcohol, formaldehyde, and phenol. However, our own research [31] and the current research did not confirm the degradation of the fiber into phenol, for which no characteristic bands from the aromatic group were identified at a wavelength of 3032 cm^−1^.

### 3.5. Evaluation of the Crystalline Structure by WAXD

**Figure 5:**
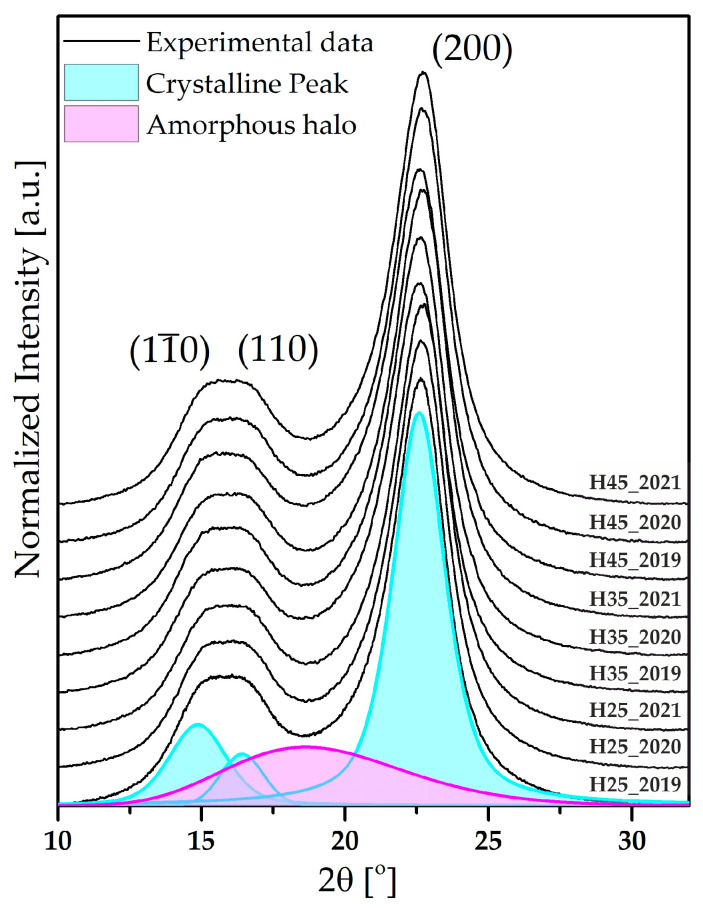
X-ray diffraction profiles of samples of hemp fibers.

The WAXD technique is used to assess the supramolecular structure of polymers in terms of crystalline phase content. In this study, X-ray radiation undergoes diffraction on ordered segments of the macromolecule (crystalline regions), while in amorphous regions, it scatters, creating an amorphous halo background. A comparison of X-ray diffraction profiles recorded for all tested variants of hemp fibers is presented in Figure 5. In the range of diffraction angles 2θ = 10–32°, characteristic diffraction peaks were located at 2θ: 14.83–15.14°; 16.38–16.72°; 22.50–22.67°, corresponding to peaks of native cellulose diffraction angles, which are 15°, 17°, and 22.7° (with Miller indices of (1$\bar{1}$0), (110), and (200)) [32,33].

The differences between the obtained WAXD profiles are inconsiderable. Therefore, the impact of drought on the crystalline structure of fibers was assessed using numerical methods. The degree of crystallinity was examined using the WAXSFIT software. The method developed by Hindeleh and Johnson allows for the decomposition of the diffraction curve into components: crystalline peaks and amorphous background [34,35]. A theoretical curve was determined to calculate the area under the curves of crystalline and amorphous components, allowing for the determination of the crystalline phase content based on the equation [36]:

(1) $$x_{k}=\frac{A_{k}}{A_{k}+A_{a}}$$

where:

*x*_*k*_—degree of crystallinity;

*A*_*k*_—area under the crystalline curve;

*A*_*a*_—area under the amorphous halo.

**Figure 6:**
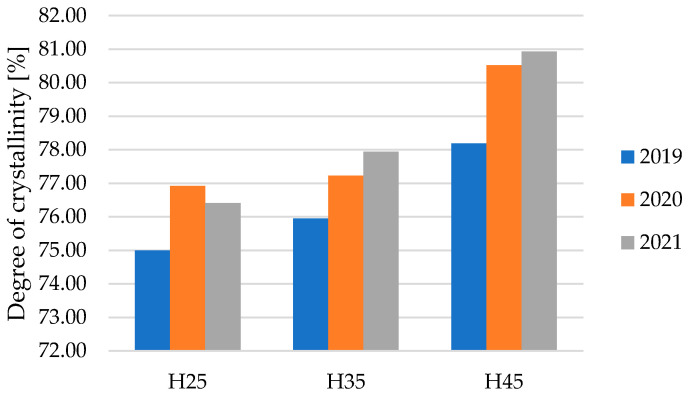
Degree of crystallinity of samples of hemp fibers.

Figure 6 presents the results of the degree of crystallinity of hemp fibers sourced from crops under controlled conditions and drought stress from a three-year experiment. The fibers exhibited a degree of crystallinity ranging from 75.00% to 80.93%. The research results showed that fibers from the control group, which were not exposed to drought stress, exhibited a higher degree of crystallinity compared to fibers subjected to water deficit. The results of the one-way analysis of variance (ANOVA) F-test (*p* = 0.0188), with the accepted level of significance (α = 0.05), indicated that the soil moisture level significantly statistically influences the degree of crystallinity of hemp fibers, where an increase in moisture content leads to an increase in the degree of fiber crystallinity. The region of fibers where macro-molecules are crystalline and aggregated exhibits specific mechanical properties related to their deformability and strength. The crystalline regions represent the most resilient parts of the fiber, with their strength surpassing several times that of the amorphous regions [37,38].

## 4. Conclusions

Global warming, characterized by a gradual rise in Earth’s average temperature, intensifies droughts worldwide, significantly affecting various sectors, particularly agriculture. Hemp, known for its versatility and drought tolerance, is still vulnerable to the negative effects of prolonged droughts. Limited water during critical growth stages can reduce yields and compromise fiber quality. The research indicates that drought notably impacts the linear density of hemp fibers, which are crucial for producing fine yarns in the textile industry, although it does not significantly affect fiber tenacity. Chemical analyses reveal that drought significantly increases the content of wax and fat in hemp fibers, substances that help create a hydrophobic layer to reduce water loss. FTIR-ATR spectrophotometric analysis showed no significant changes in the molecular structure of hemp fibers due to drought. However, thermal analysis revealed that higher soil moisture capacity lowers the decomposition temperature of hemp fibers. Regardless of soil moisture, pyrolysis of hemp fibers releases gases like CO_2_ and methane. WAXD analysis showed that fibers from plants not exposed to drought have a higher degree of crystallinity than those subjected to water deficiency. These findings highlight the challenges the textile and industrial sectors may face in maintaining consistent quality and meeting consumer demands. To address drought’s impact on hemp cultivation, adaptive strategies, sustainable water management practices, and global climate change mitigation efforts are essential.

## Figures and Tables

**Table 1:** List of symbols of Bialobrzeskie hemp fibers grown under specific soil moisture conditions.

Symbol	Variety of Hemp	The Level of Drought Stress
H25	Bialobrzeskie Hemp	25% field water capacity of the soil
H35		35% field water capacity of the soil
H45		45% field water capacity of the soil

**Table 2:** Analysis of the type of vibrations and functional groups for range of wavenumbers of FTIR-ATR fiber spectra.

Range of Wavenumbers	Type of Vibration	Functional Group	Occurrence
3336 cm^−1^	stretching	OH	cellulose, hemicellulose, lignin, pectin
3282 cm^−1^			
2917 cm^−1^	stretching	CH, CH_2_	cellulose, hemicellulose, lignin, pectin, wax, and fat
2849 cm^−1^			
1732 cm^−1^	stretching	C=O	lignin, pectin, wax, and fat
1629 cm^−1^	stretching	OH	adsorbed water
1423 cm^−1^	stretching	C=C	lignin
1315 cm^−1^	scissoring (bending)	CH_2_	cellulose, hemicellulose
1241 cm^−1^	deforming	OH	cellulose, hemicellulose
1023 cm^−1^–1051 cm^−1^	bending	COC	cellulose, hemicellulose, pectin
888 cm^−1^	stretching	β-glycosidic linkage	cellulose, hemicellulose, pectin

**Table 3:** Thermal properties of hemp fibers.

Fibers	Decomposition Temperature [°C]						Residual Mass at T_700_ °C	
	DTG		T_10%._		T_60%._			
	°C	SD	°C	SD	%	SD	%	SD
2019								
H25	364.10	0.12	290.69	13.92	367.36	0.64	14.91	0.24
H35	363.20	0.14	289.70	5.86	366.44	0.17	14.94	0.16
H45	362.91	0.45	291.51	3.15	366.15	0.40	15.04	0.19
2020								
H25	363.85	0.16	284.06	10.15	367.04	0.45	15.22	0.26
H35	364.77	0.29	284.23	14.08	367.94	0.84	14.85	0.57
H45	364.28	0.42	294.87	10.90	368.15	0.50	15.22	0.62
2021								
H25	363.99	0.12	282.00	11.85	367.51	0.48	14.86	0.96
H35	364.87	0.19	295.81	12.89	368.69	0.66	15.58	0.36
H45	364.77	0.09	291.92	11.37	368.97	0.34	16.11	0.27

**Table 4:** Analysis of the type of vibrations and functional groups for range of wavenumbers of FTIR spectra of released gases during the pyrolysis of hemp fibers from 2019–2021.

Year of Experiment	Temperature	Range of Wavenumbers	Type of Vibration	Functional Group
2019–2021	100 °C	3747–3736 cm^−1^	stretching	OH
		1511–1509 cm^−1^	stretching	OH
	300 °C	3740–3567 cm^−1^	stretching	OH
		2983–2732 cm^−1^	stretching	CH_3_, CH_2_, CH
		2362–2361 cm^−1^	stretching	CO_2_
		2195–2183 cm^−1^	stretching	CO
		1773–1769 cm^−1^	stretching	C=O
		1379–1376 cm^−1^	deforming	CH_3_
		1180–1077 cm^−1^	stretching	C-O
		673–673 cm^−1^	stretching	CO_2_
	365 °C	3740–3566 cm^−1^	stretching	OH
		2982–2731 cm^−1^	stretching	CH_3_, CH_2_, CH
		2364–2362 cm^−1^	stretching	CO_2_
		2189–2184 cm^−1^	stretching	CO
		1749–1745 cm^−1^	stretching	C=O
		1384–1377 cm^−1^	deforming	CH_3_
		1085–1084 cm^−1^	stretching	C-O
		673–670 cm^−1^	stretching	CO_2_
	450 °C	3737–3569 cm^−1^	stretching	OH
		3022–2928 cm^−1^	stretching	CH_4_, CH
		2362–2361 cm^−1^	stretching	CO_2_
		2188–2182 cm^−1^	stretching	CO
		1755–1751 cm^−1^	stretching	C=O
		1112–1053 cm^−1^	stretching	C-O
		673–670 cm^−1^	stretching	CO_2_

## Data Availability

Raw data are available upon request.

## Supplementary Materials

The following supporting information can be downloaded at: https://www.mdpi.com/article/10.3390/ma17174198/s1.
