# Supplementary material for: Assessing the Impact of Drought Stress on Hemp (Cannabis sativa L.) Fibers
Source: Materials (Basel). 2024 Aug 24;17(17):4198. doi: 10.3390/ma17174198 (PMC11396084; doi:10.3390/ma17174198)
Supplement: Supplementary file 1 [file materials-17-04198-s001.zip › descriptions.pdf]

S1: The FTIR-ATR spectra of hemp fibers

S2: FTIR spectra of released gases during the pyrolysis of hemp fibers, from 2019

S3: FTIR spectra of released gases during the pyrolysis of hemp fibers, from 2020

S4: FTIR spectra of released gases during the pyrolysis of hemp fibers, from 2021
