# Supplementary figures and images for: Assessing the Impact of Drought Stress on Hemp (Cannabis sativa L.) Fibers
Source: Materials (Basel). 2024 Aug 24;17(17):4198. doi: 10.3390/ma17174198 (PMC11396084; doi:10.3390/ma17174198)

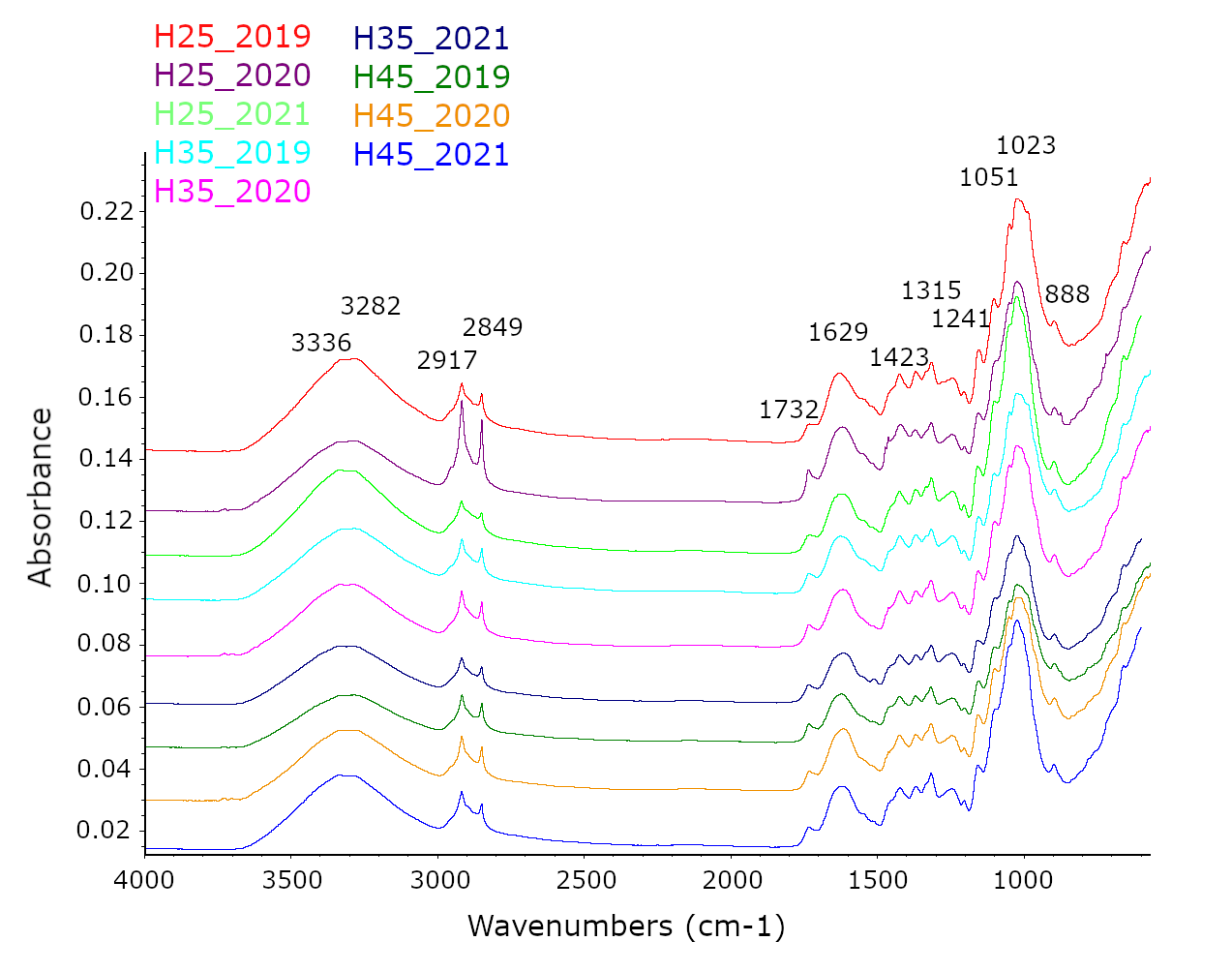

Supplement: Supplementary file 1 [file materials-17-04198-s001.zip › supplementary file 1.png]

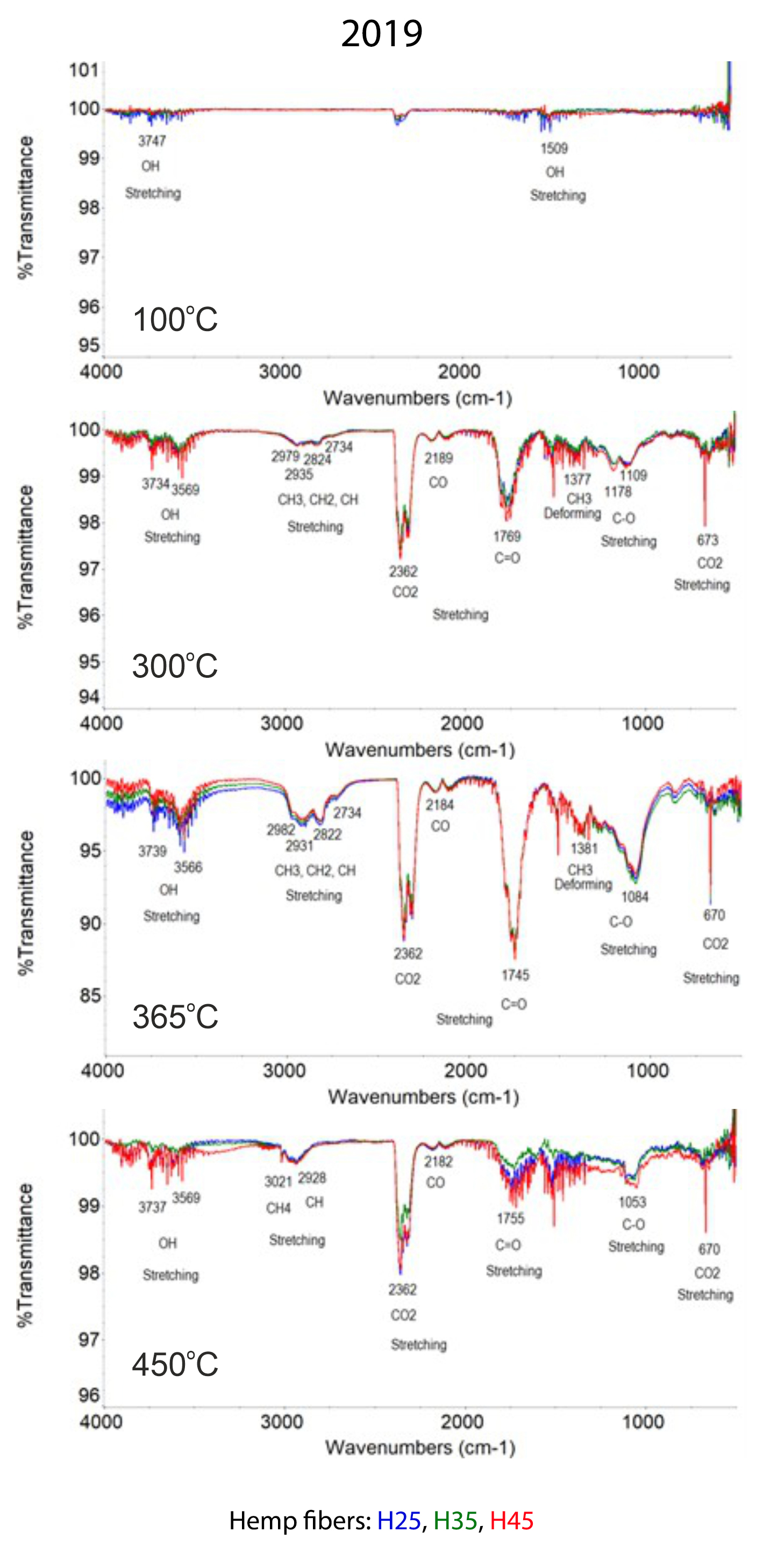

Supplement: Supplementary file 1 [file materials-17-04198-s001.zip › supplementary file 2.jpg]

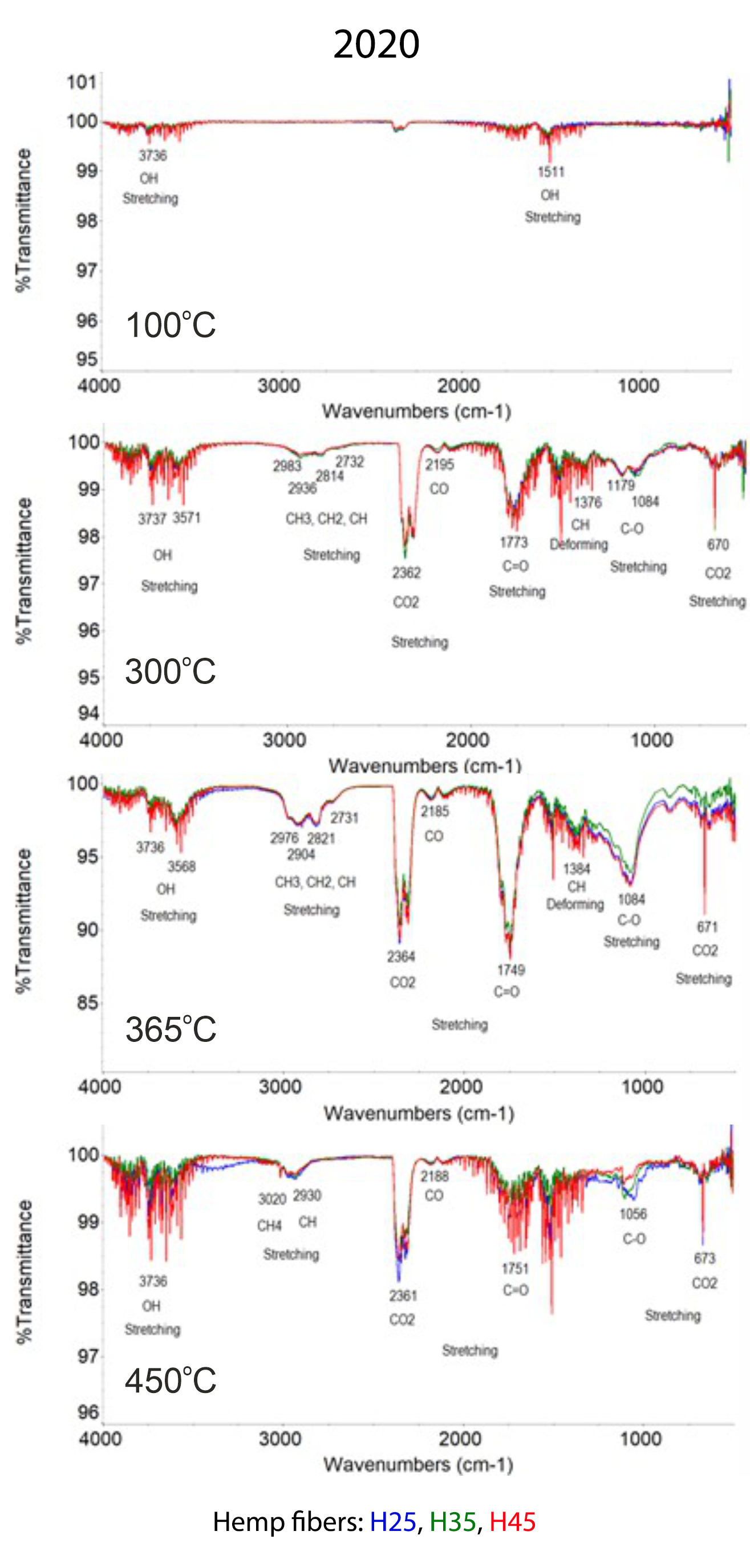

Supplement: Supplementary file 1 [file materials-17-04198-s001.zip › supplementary file 3.jpg]

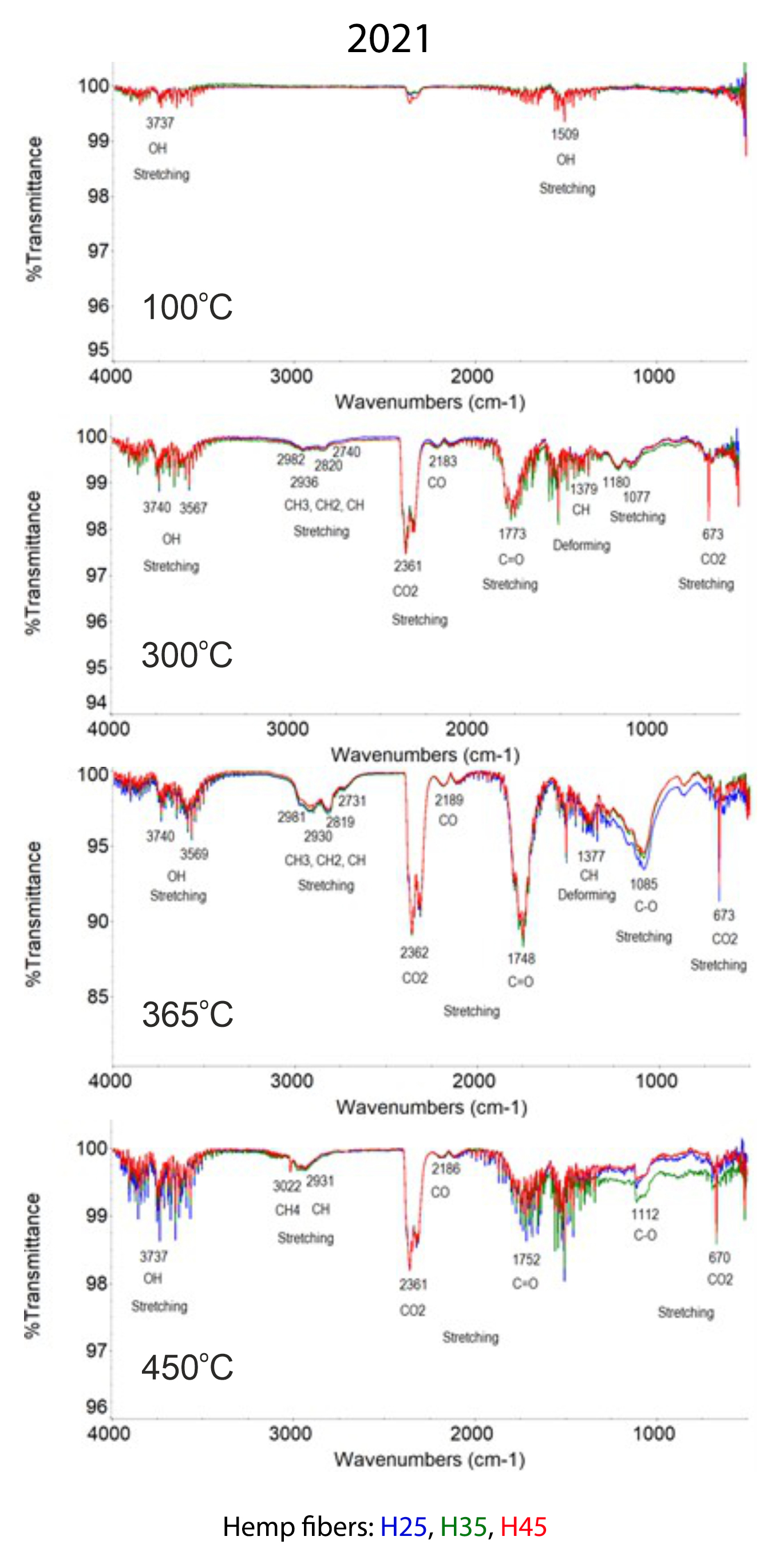

Supplement: Supplementary file 1 [file materials-17-04198-s001.zip › supplementary file 4.jpg]
